# Detection of the fungicide transformation product 4-hydroxychlorothalonil in serum of pregnant women from Sweden and Costa Rica

**DOI:** 10.1038/s41370-023-00580-8

**Published:** 2023-07-20

**Authors:** Annette M. Krais, Berna van Wendel de Joode, Emelie Rietz Liljedahl, Annelise J. Blomberg, Anna Rönnholm, Marie Bengtsson, Juan Camilo Cano, Jane A. Hoppin, Margareta Littorin, Christel Nielsen, Christian H. Lindh

**Affiliations:** 1 Division of Occupational and Environmental Medicine, Department of Laboratory Medicine, Lund University, Lund, Sweden; 2 Infants’ Environmental Health Study (ISA), Central American Institute for Studies on Toxic Substances (IRET), Universidad Nacional de Costa Rica, Heredia, Costa Rica; 3 Center for Human Health and the Environment, North Carolina State University, Raleigh, NC USA; 4 Department of Biological Sciences, North Carolina State University, Raleigh, NC USA; 5 Divison of Clinical Pharmacology, Pharmacy and Environmental Medicine, Department of Public Health, University of Southern Denmark, Odense, Denmark

**Keywords:** Chlorothalonil, Fungicide, Exposure biomarker, Human biomonitoring, Aerial spraying, Pesticides

## Abstract

**Background:**

4-hydroxychlorothalonil (HCT, R182281), a transformation product of the fungicide chlorothalonil, was recently identified in human serum and breast milk. There are indications that HCT may be more toxic and environmentally persistent than chlorothalonil.

**Objective:**

Our aim was to investigate serum concentrations of HCT in pregnant women in Sweden and Costa Rica.

**Methods:**

We developed a quantitative analytical method for HCT using liquid chromatography tandem mass spectrometry. We measured HCT in 1808 serum samples from pregnant women from the general population in Sweden (1997–2015) and in 632 samples from 393 pregnant women from an agricultural population in Costa Rica (2010–2011). In Swedish samples, we assessed time trends and investigated seasonality. In the Costa Rican samples, we evaluated variability between and within women and explanatory variables of HCT concentrations.

**Results:**

HCT was detected in all serum samples, and the limit of detection was 0.1 µg/L. The median HCT concentration in the Swedish samples was 4.1 µg/L (interquartile range [IQR] of 2.9 − 5.8 µg/L), and 3.9 times higher in the Costa Rican samples (median: 16.1 µg/L; IQR: 10.6 − 25.0 µg/L). We found clear seasonal variation with higher concentrations in the first half of each year among Swedish women. In the Costa Rican study, women working in agriculture and living near banana plantations had higher HCT concentrations, whilst higher parity and having a partner working in agriculture were associated with decreased HCT, and no clear seasonal pattern was observed.

**Impact statement:**

For the first time, this study quantifies human exposure to the fungicide chlorothalonil and/or its transformation product 4-hydroxychlorothalonil (HCT, R182281) and finds higher serum concentrations in women from a tropical agricultural setting as compared with women from the general population in Sweden.

## Introduction

**Fig. 1:**
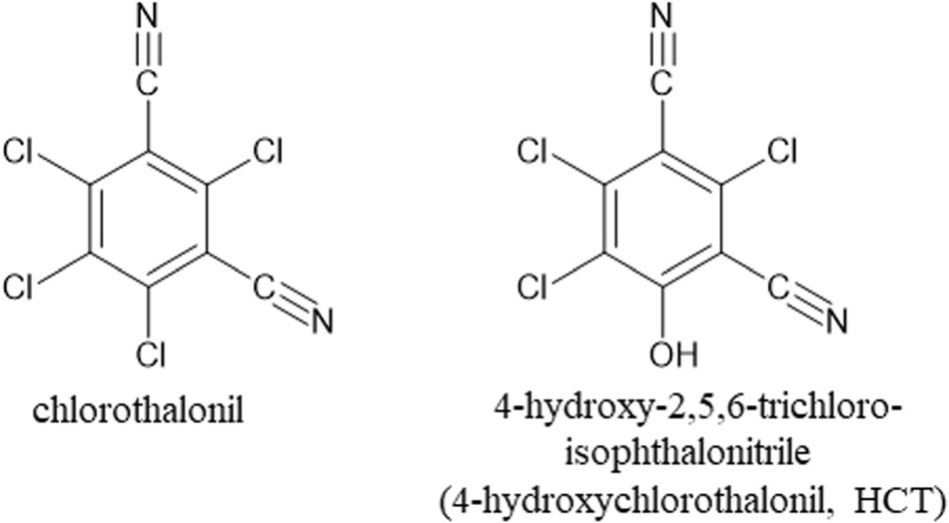
Chemical structures of chlorothalonil and 4-hydroxychlorothalonil (HCT).

Non-targeted screening approaches have revealed the presence of 4-hydroxy-2,5,6-trichloroisophthalonitrile (4-hydroxychlorothalonil, HCT), a transformation product of the fungicide chlorothalonil in human blood [1] and in breast milk [2, 3]. However, no quantitative analysis of chlorothalonil or its transformation products in human samples has yet been reported. Fig. 1 shows the chemical structures of both, chlorothalonil and 4-hydroxychlorothalonil (HCT).

Chlorothalonil is an organochlorine fungicide developed in the United States in 1966 [4] that has both agricultural and household uses. It is applied to control diseases in many fruits, vegetables, and other agricultural crops [5–7], and it is also used as paint additive and wood preservative [4, 5]. In 2019, the European Union banned the use of chlorothalonil in agriculture due to health and environmental concerns [8], while in Sweden, the use of chlorothalonil in agriculture has been discontinued since 1991 [9]. Chlorothalonil was still in use as a paint additive and wood protective in Sweden until its EU-wide ban in 2011 [9, 10]. However, while no chlorothalonil was found in a national screening of environmental samples in 2008, performed in paint factories, landfills, paint storage sites, and boat marinas in Sweden [9], chlorothalonil transformation products were not explored in this monitoring study.

In Central America, chlorothalonil is widely used in agriculture, including applications by light aircraft on bananas grown for export purposes [11]. This agricultural use may result in environmental and occupational exposures and could affect human health. In 2020, chlorothalonil was detected in 50% of the 48 passive air samples obtained from 12 schools, and in 60% of 15 active air samples obtained from 4 schools near banana plantations in Matina County, Limon, Costa Rica [11].

Chlorothalonil has been classified by the International Agency for Research on Cancer (IARC) as possibly carcinogenic to humans, based on results from animal studies (Group 2B) [7]. Yet, although studies in human beings are lacking, a carcinogenicity category 1B (Carc. 1B, H350, ‘may cause cancer’) has been proposed following current classification criteria [6]. Furthermore, occupational exposure to chlorothalonil during its production and during application may cause occupational asthma, allergic contact dermatitis, or anaphylaxis [5, 7, 12–15]. Reports of acute toxicological effects from non-occupational exposures are rare [15–17].

Chlorothalonil contaminates water supplies adjacent to agricultural areas where it is applied [18, 19], and it strongly sorbs to soil and sediment [20]. Chlorothalonil can be degraded in soil and water by aerobic or anaerobic microbes [20], or by ultraviolet (UV) light from solar irradiation [21] with a half-life of one to 48 h [22]. Many different transformation products are formed [6], but the principal transformation product of chlorothalonil in environmental samples is HCT, also listed as transformation product R182281 or SDS-3701 [5]. Several studies have detected HCT in soil [23–25] and water [22, 26].

There are indications that several chlorothalonil transformation products may be more persistent, mobile, or toxic than chlorothalonil [4, 9, 27]. HCT has been found to be more persistent and less mobile in soil compared to chlorothalonil [6], more toxic to birds but less toxic to aquatic organisms [4, 5], and only moderately toxic to mammals [5, 6]. No reports exist on HCT toxicity in humans, and there is a lack of knowledge about human metabolism of chlorothalonil in general [6].

Our primary aim was to investigate HCT serum concentrations in two cohorts of pregnant women, a general population sample from Sweden and a sample of women from a banana-growing region in Costa Rica. We developed a quantitative method using liquid chromatography-tandem mass spectrometry (LC-MS/MS). The population from Sweden was included as a general population sample with assumed low probability of exposure, while the population from Costa Rica was expected to be exposed, either occupationally or by living in proximity to banana plantations where chlorothalonil was being applied by aerial spraying. To characterize exposure patterns, we assessed temporal trends (1997–2015) and seasonal variation in serum concentrations in samples from Sweden, and contrasted concentrations in the Swedish population to those in the Costa Rican population. In the Costa Rican samples, we evaluated whether age, parity, gestational age at sampling, seasonal variation, occupational characteristics, and residential distance to plantations on which bananas were grown for export purposes partly explained serum HCT. In addition, we estimated variability between and within women and compared HCT concentrations among women with repeated samples.

## Material and methods

### Quantitative analysis of HCT in serum

Samples were analyzed at the Department of Occupational and Environmental Medicine, Lund University, Sweden. The analytical method was adapted from Norén et al. [28] and is described in detail in the Supplementary Information.

#### Sample preparation

10 µL of β-glucuronidase solution and 10 µL of 1 M ammonium acetate buffer (pH 6.5) were added to 100 µL of serum samples in a 96-well plate, and mixed for 90 min at 37 °C. Thereafter, 25 µL of acetonitrile or standard solution was added to each sample. Proteins were precipitated with 200 µL of acetonitrile, and samples were mixed and centrifuged at 2600 × *g* for 10 min. The supernatant was transferred to a new 96-well plate and centrifuged at 3000 × *g* for 10 min prior to analysis. Blank controls (100 µL MilliQ water) and quality control samples were analyzed with every analytical batch in duplicates. The authors are aware of the fact that the hydrolysis step with glucuronidase has not been optimized.

#### LC-MS/MS instrumentation and conditions

An aliquot of 5 µL was injected onto the analytical column (Gemini NX-C18, Phenomenex, Torrance, CA, USA). The mobile phases were 5 mM ammonium acetate in water (A) and methanol (B). A Genesis Lightning C18 column (Avantor, VWR International, Lutterworth, UK) was used before the injector to filter the mobile phases from contaminating substances.

Quantitative analysis was conducted using a liquid chromatography system (UFLCXR, Shimadzu Corporation, Kyoto, Japan; LC-MS/MS) coupled to a triple quadrupole linear ion trap mass spectrometer equipped with a TurboIonSpray source (QTRAP® 5500, AB Sciex, Framingham, MA, USA). The MS analyses were carried out using selected reaction monitoring (SRM) in negative ion mode. The quantitative analysis of HCT was performed using the transitions m/z 245/175 and 245/35 (Table S1). Chlorothalonil was not analyzed as results from previous studies using untargeted analysis approaches have revealed the presence of HCT, but not chlorothalonil, in human serum [1]. All data acquisition was performed using Analyst 1.7.2 software and data processing was performed using Multiquant 3.0.1 (AB Sciex, Framingham, MA, USA).

### Study population: Sweden

We used biobanked samples (*n* = 1808) from the Swedish Rubella Screening Program [29–31] that started in 1989 and contains >250,000 maternal serum samples. Since 1989, almost all pregnant women in Scania County have participated in serological screening for viral infections and rubella immunity during weeks 12–14 of pregnancy [29]. Samples were originally retrieved for the Autism and Prenatal Endocrine Disruptors (APED) study, with the aim to determine the impact of prenatal exposure to multiple classes of endocrine disruptors, including organochlorine pesticides and autism risk. Prenatal samples from mothers of autism cases and matched controls were selected from the biobank (storage at −80 °C) and analyzed randomized. The study was approved by the Swedish Ethical Review Authority (DNR 2015/221). Seasonal distributions of exposure might have changed over the years as a result of shifts in consumption patterns of imported food. Age distributions of the study population are presented in Table 1. In the current study, one sample per woman was analyzed (*n* = 1808) for HCT.

**Table 1:** Cohort characteristics, displayed as *N* (%), and 4-hydroxychlorothalonil (HCT) serum concentrations (µg/L) in the Swedish and Costa Rican studies: median values with interquartile range (IQR), mean values with standard deviation (SD).

	Sweden (1 sample per woman)	Costa Rica	
		Sample 1	Sample 2
N (samples)	1808	393^a^	239^a^
Years	1997–2015	2010–2011	2010–2011
Type of Population	General	Agricultural	Agricultural
Maternal age^b^	<25 years: 277 (15.3%) 25–34 years: 1161 (64.3%) 35+ years: 368 (20.4%)	<18 years: 70 (17.8%) 18–24 years: 188 (47.8%) 25–34 years: 105 (26.7%) 30+ years: 30 (7.6%)	45 (18.8%) 112 (46.9%) 63 (26.4%) 19 (7.9%)
Parity (*n* > 1%)^c^	929 (51%)	247 (63%)	148 (62%)
Sampling time	Early pregnancy (weeks 12 − 14)	1st trimester 75 (19%) 2nd trimester 218 (56%) 3rd trimester 100 (25%)	1st trimester 1 (0%) 2nd trimester 75 (32%) 3rd trimester 163 (68%)
Sampling Period		May 2010 - Aug 2011	June 2010 - Nov 2011
Relatively dry season (Feb-Apr and Sept-Oct)		176 (44.8%)	121 (50.6%)
Married/living as married		300 (76.3%)	177 (74.1%)
Woman working in agriculture		32 (8.1%)	21 (8.8%)
Partner working in agriculture^d^		230 (58.5%)	140 (58.6%)
Living distance to banana plantations (meters) Median (IQR)		214 (50–557)	197 (49–457)
HCT serum concentrations (µg/L)			
Median (IQR)	4.1 (2.9 − 5.8)	16.1 (10.6−25.0)	15.1 (9.9−23.0)
Range	0.2 − 38	0.5 − 136	2.0 − 97.1

^a^55 samples missing from the original cohort (*n* = 451); 393 had at least one serum sample analyzed for HCT, and for *n* = 239 women a second sample was analyzed; thus *n* = 632 samples in total.

^b^Information on maternal age not available for 2 samples in the Swedish cohort.

^c^Information on parity not available for 8 samples in the Costa Rican cohort and imputed with the number of woman’s children living with her.

^d^For women without a partner (*n* = 11) missing value was replaced with ‘not working in agriculture’.

### Study population: Costa Rica

The Infants’ Environmental Health Study (Infantes y Salud Ambiental, ISA) is a community-based birth cohort coordinated by the Universidad Nacional, Costa Rica. The study examines the potential health effects of pesticides on the participating mothers and their children [32–34]. The study is situated in Matina County, Costa Rica, an area with extensive banana production for export purposes [35, 36]. Between March 2010 and June 2011, 451 pregnant women (98% response rate) were enrolled. About half of the women lived less than 200 meters (percentile p25 = 49, p75 = 565) from banana plantations (Fig. S4) [31]. Study activities were approved by the Scientific Ethics Committee of the Universidad Nacional in Costa Rica (CECUNA-11-2009). More study details are published elsewhere [35, 36]. In the current study, we analyzed HCT in 632 serum samples from 393 pregnant women: one first serum sample (*n* = 393) at mean gestational age (SD) = 21 ( ± 7.9) weeks, and a second serum sample (*n* = 239) later during pregnancy (mean gestational age at sampling 29 ( ± 6.0) weeks). Serum samples were stored at −20 °C and shipped to Sweden on ice. Age distributions of the whole study population with serum samples (*n* = 393), as well as other characteristics are presented in Table 1.

### Statistical data analysis

Statistical analysis was performed using R Statistical Software (version 4.2.1; R Foundation for Statistical Computing, Vienna, Austria). In the Swedish cohort, we investigated long-term temporal, including seasonal, trends in HCT serum concentrations. We tested for seasonal differences using non-parametric tests, due to strong right-skewed distributions of concentrations. We defined seasons as winter (December, January, February), spring (March, April, May), summer (June, July, August) and autumn (September, October, November). We tested for an overall effect of season using a Kruskal Wallis Test and for differences between seasons using Wilcoxon-Mann-Whitney tests with a Bonferroni correction for multiple comparisons. We then fitted a linear regression model for HCT concentrations by calendar time (in days). HCT concentrations were ln-transformed to ensure normality of the residuals. The model also controlled for seasonality using a sine and cosine term with a period of 365.25 days, which allows for a seasonal pattern following a cosine function with a period of one year and a variable horizontal and amplitude shift [37]. Finally, we investigated determinants of serum concentrations by regressing ln-HCT concentrations on categorical variables for age (tertiles: <28; 28–32; ≥32) and parity (0;1; ≥2).

In the Costa Rican population, we used descriptive statistics and distributional plots to examine all variables. The Costa Rica Caribbean has a hot humid climate, with similar climatological conditions all year round. Bananas are being grown, and pesticides being sprayed, during the whole year. The somewhat dryer months are February-April and October-November. As HCT concentrations were right-skewed, we used the Wilcoxon signed-rank test for paired samples to test whether concentrations of HCT were different between the first and second sample in women with both samples. We subsequently ln-transformed HCT prior to statistical modeling to normalize residuals. We applied a mixed-effects linear regression model with a random intercept for each participant to estimate variability between and within women and calculated the intraclass correlation coefficient (ICC) of HCT.

We categorized maternal age into four groups with an equal number of observations (using 25th, 50th, 75^th^ percentiles as cut-off points), and parity and residential distance to banana plantations into three groups with an equal number of observations (using 33^rd^, 66^th^ percentiles as cut-off points). We then investigated potential predictors of the ln-HCT concentrations in the initial serum sample using simple linear regression models for the following variables obtained at enrolment: 1) season at sampling [relatively dry (February-April or October-November) or wet (other months) season], 2) woman’s age (<19, ≥19–21, ≥22–27, ≥28 years), 3) parity (0, 1, ≥2), 4) gestational age at sampling (weeks), 5) woman works in agriculture (yes/no), 6) partner works in agriculture (yes/no), 7) residential distance to banana plantations at enrolment (< 90, ≥90–372, ≥373 meters). We subsequently ran separate multiple linear regression models for ln-HCT concentrations in the first and second serum samples and included the seven co-variables a priori. We calculated the geometric mean of the intercept by exp(β), and percentage change of variables by [exp(β)-1] * 100. For categorical variables this reflect the percentage change as compared to the reference category ( = intercept) and for continuous variables the percent change for each 1-unit increase. We presented 95%CI of these estimates to show precision of the effect size. Because of the exploratory nature of our analysis, we focused on patterns of associations instead of statistically significant findings (e.g. 95% confidence intervals that do not include zero). We checked if models met assumptions statistical criteria of validity (normality of residuals, lack of multicollinearity, homoscedasticity, Cook’s distance).

## Results

In this study, samples from 2201 women were included: 1808 from the APED study in Sweden and 393 from the ISA study in Costa Rica. For 239 of the 393 women from the Costa Rican study we obtained a second sample (n_total_ = 632). Population characteristics are summarized in Table 1.

### Method validation of HCT

The limit of detection (LOD) was determined as 0.1 µg/L and was defined as three times the standard deviation of the concentration corresponding to the peak at the same retention time as HCT in the chemical blanks (*n* = 128). Equally, the limit of quantification (LOQ) was determined as 0.4 µg/L and defined as ten times the standard deviation. A typical chromatogram of a real serum sample is shown in Fig. S2. The results of the quality control samples were used to calculate the between-run precision of the method determined as the coefficient of variation (CV). The quality control samples were analyzed with every analytical batch in duplicates. The CV at 3 μg/L was 14%, and at 5 μg/L it was 13% (*n* = 39) (Table S2). The between-batch precision was determined from comparing duplicate analyses of samples expressed as the CV. The CV at 7 μg/L was 7% (*n* = 238), at 15 μg/L it was 7% (*n* = 239), and at 32 μg/L it was 11% (*n* = 239) (Table S2). More details are described in the Supplementary Information.

### Regional distribution of HCT serum levels

In both the Swedish and Costa Rican cohorts, HCT was detected (LOD = 0.1 µg/L) in all serum samples (*n* = 1808 and *n* = 632, respectively). The Swedish serum concentrations ranged from 0.2 to 38 µg/L with a median concentration of 4.1 µg/L (IQR: 2.9 − 5.8 µg/L), while the Costa Rican serum concentrations ranged from 0.5 to 136 µg/L, with a median concentration of 16.1 µg/L (IQR: 10.6 − 25.0 µg/L) for the first serum samples (*n* = 393), which is 3.9 times higher than the median in the Swedish samples (Table 1). The median concentration in the second serum samples was somewhat lower at 15.1 µg/L (IQR: 9.9 − 23.0 µg/L, *p* < 0.001).

### Temporal trends and seasonality in the Swedish cohort

To understand how exposure has changed over time, we assessed potential temporal trends in serum concentrations in samples from Sweden. Concentrations by year showed no clear pattern over time (Table 2 and Fig. S3). This was confirmed by the linear regression analysis of HCT serum concentrations (µg/L) by time (estimated change per year of −0.34% (95% CI: −1.01, 0.33)).

**Table 2:** Descriptive statistics of 4-hydroxychlorothalonil (HCT) serum concentrations (µg/L) in 1808 pregnant Swedish women and in 393 women from Costa Rica.

Sweden (*n* = 1806)				
Year	*N*	Median (IQR)	P05–P95	Range I
1997	5	3.7 (2.9–3.7)	2.5–3.8	2.4–3.8
1998	31	3.5 (2.1–4.4)	1.8–5.9	0.88–10
1999	38	3.7 (2.4–4.5)	1.3–6	1.1–6.4
2000	40	3.2 (2.1–4.6)	1.4–6.7	1.1–11
2001	77	3.7 (2.6–4.9)	1.6–9	0.99–14
2002	84	3.8 (3.1–4.5)	2.1–7.9	1.9–21
2003	145	4.3 (3.2–5.6)	1.8–7.5	1.2–10
2004	111	4.5 (3.2–5.7)	1.5–8.3	0.72–14
2005	137	4.6 (3.3–6.6)	1.8–11	0.17–19
2006	151	4.5 (3.4–6.5)	1.9–8.7	0.97–14
2007	153	5.2 (3.9–6.8)	2.9–10	0.87–21
2008	188	4.7 (2.8–7.3)	1.4–12	0.28–17
2009	189	3.6 (2.4–5.4)	1.4–10	0.62–38
2010	160	3.7 (2.5–5.2)	1.3–8	0.42–18
2011	142	3.4 (2.6–5.1)	1.6–8.2	0.27–14
2012	88	3.7 (2.7–5)	1.8–7.7	1.2–16
2013	62	4.3 (2.9–5.5)	1.7–8.2	0.58–11
2014	5	4.8 (3.2–5.6)	2.1–8.3	1.8–9.0
2015	2	5.3 (4.1–6.6)	3.1–7.5	2.9–7.8

Costa Rica (*n* = 393)				
2010–2011	393 (visit 1)	16.1 (10.6–25.0)	4.9–42.8	0.50–136
2010–2011	239 (visit 2)	15.2 (10–23.1)	6.6–34.2	2.0–97.1

**Fig. 2:**
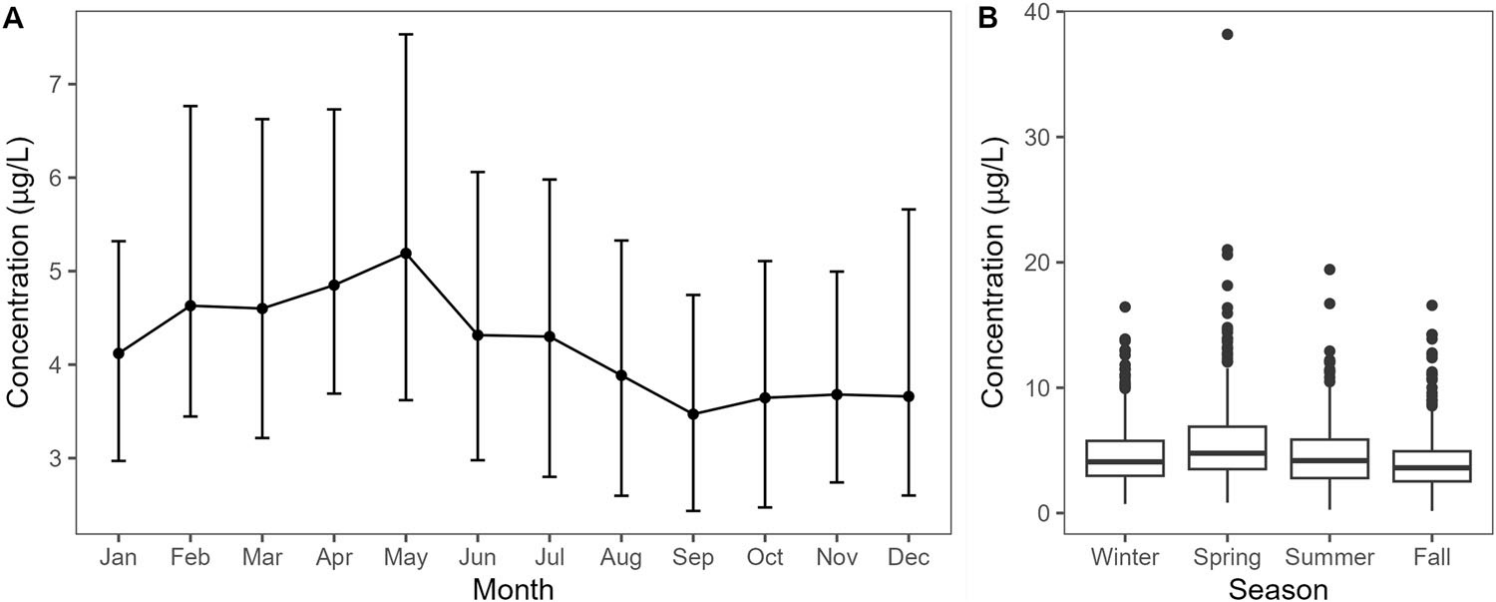
Distribution of 4-hydroxychlorothalonil (HCT) concentrations by month and season in the Swedish population. Median and IQR values by month (**A**) and boxplots of concentrations by season (**B**).

Serum concentrations of HCT changed by month, with higher concentrations in the first six months of the year, peaking in May, and then decreasing over the second half of the year (Fig. 2A, B). There was an overall difference in concentration across all four seasons, and pairwise comparisons showed significant differences between all four seasons except when comparing winter to summer (Table S3).

### Between-subject variability and difference between first and second sample in Costa Rica population

The distribution of 4-OH-CHT in serum during pregnancy of visit 1 (*n* = 393) and visit 2 (*n* = 239) and the residential distance to banana plantations (*n* = 393) are shown in Fig. S4. The mixed-effects model of the repeated serum samples had an ICC value of 0.81, indicating that most of the model variability was due to between-subject variation. Results from paired samples Wilcoxon test among women with both a first and second serum sample (*n* = 239) showed 4-OH-CHT concentrations from first samples were higher than second samples (difference in medians=2.0 µg/L, 95CI%: 1.4, 2.6, *p* < 0.001).

### Variables associated with HCT exposure

Table 3A shows the determinants of HCT concentrations in Swedish pregnant women. We found that increasing parity was associated with decreasing serum levels whereas increasing age was associated with increasing serum levels.

**Table 3:** Results from multivariate regression models presenting percentage change in 4-hydroxychlorothalonil (HCT) serum concentrations associated with age and parity among pregnant women from Sweden (A), and associated with sociodemographic, pregnancy, environmental and pregnancy variables, among pregnant women from Costa Rica (**B**)^a^.

A	
Variables	*n* = 1806 R^2^ = 0.09
Intercept	
Geometric mean (95% CI) HCT (µg/L)^a^	4.3 (4.1, 4.5)***
	***% Change HCT (95% CI)***
**Maternal age (years) (tertiles)**	
≥28 – 32 vs. <28	19 (11, 26)***
≥32 vs. <28	18 (11, 26)***
**Parity**	
1 vs. 0	−27 (−31; −23)***
≥2 vs. 0	−31 (−36, −26)***

B		
Variables	First samples, *n* = 393 R^2^ = 0.11, R^2^-adj = 0.08	Second samples, *n* = 239 R^2^ = 0.12, R^2^-adj = 0.08
Intercept		
Geometric mean (95% CI) HCT (µg/L)^a^	16.7 (12.7, 22.1)***	21.5 (13.9, 33.3)***
	***% Change HCT (95% CI)***	***% Change HCT (95% CI)***
**Maternal age (years) (tertiles)**		
≥19 – 21 vs. <19	22 (0, 47)*	37 (8, 72)**
≥22 – 27 vs. <19	55 (25, 92)***	43 (11, 84)**
≥28 vs <19	67 (28, 117)***	41 (3, 92)*
**Parity**		
1 vs. 0	−12 (27, 6)	−10 (−28, 11)
2 vs. 0	−29 (−43, −10)**	−24 (−42, −1)*
**1 week increase in gestational age**	−0.45 (−1.28, 0.39)	−1.06 (−2.33, 0.22)
**Residential distance to banana plantations (m) (tertiles)**		
≥90 – 372 vs. <90	4 (−12, 23)	−2 (−18, 18)
≥373 vs. <90	−19 (−31, −5)*	−27 (−40, −11)**
**Woman works in agriculture**	27 (−1, 63)	20 (−9, 59)
**Partner works in agriculture**	−17 (−28, −5)**	−15 (−27, 0)*
**Relatively dry season (Feb-Apr or Oct-Nov)**	13 (−1, 29)	1 (−13, 18)

^a^We calculated the geometric mean of the intercept by exp(β), and percentage change of variables by [exp(β)-1]*100. For categorical variables this reflects the percentage change as compared to the reference category ( = intercept) and for continuous variables the percent change for each 1-unit increase. ***p < 0.001; **p < 0.01; *p < 0.05.

Results from the multivariate linear regression analysis of HCT concentrations in the first and second samples of the Costa Rican cohort and potential predictors are presented in Table 3B. For results from simple regression analysis see Table S4. Results for our multivariate models were similar for Sample 1 and Sample 2 (Table 3B). For Sample 1, the model found that women who were older had increased HCT [e.g. a 67% (95% CI: 28%, 117%) increase for the upper quartile as compared to the lowest quartile], whilst parity ≥2 was associated with lower HCT compared to parity=0 [a −29% (95% CI: −43%, −10%) decrease]. Women with increased gestational age tended to have lower HCT, but estimate was imprecise reflected by the rather broad confidence interval. Women who lived at a longer distance from banana plantations (upper tertile ≥ 393 meters) had lower HCT [−19%, 95% CI: −31, −5] as compared to women living more nearby (lower tertile, <90 meters). Pregnant women who worked in agriculture generally showed an increased serum HCT of 27% (95% CI −1%, 63%), but having a partner who worked in agriculture was associated with lower HCT (−17% (95% CI −28%, −5%). For the first, but not second, serum samples, we observed slightly higher HCT among the relatively dry season. For the other variables HCT concentrations from the second samples showed similar associations as the first samples.

## Discussion

This is the first study to quantify the chlorothalonil transformation product HCT in human serum. While non-targeted screening approaches have revealed the presence of HCT in human blood [38] and in breast milk [2, 3], no quantitative analytical methods for chlorothalonil or its transformation products have hitherto been reported.

The high detection frequency (100% >LOD) of HCT in our samples (collected 1997 − 2015 in Sweden and 2010 − 2011 in Costa Rica) indicates exposure of chlorothalonil or its transformation product HCT in these populations. The lack of time trends in the Swedish samples suggests that chlorothalonil (or HCT) exposure has been constant over 25 years and/or that sampling storage may not affect the measured concentrations. However, thorough tests are needed to investigate stability of HCT in human serum. Furthermore, the relatively high intra-class correlation coefficient of HCT among the Costa Rica women is consistent with the persistent character of chlorothalonil and HCT.

Our data shows that, although exposure levels were higher in the Costa Rican cohort, the pattern of decreasing serum levels with increasing parity and increasing serum levels with increasing age is found in both datasets. This strengthens the argument of the bioaccumulative potential of HCT and suggests that HCT is transferred from mother to child during pregnancy and/or breastfeeding.

A limitation of the Swedish study was that we did not obtain repeated samples from the same women, which limits a precise comparison in time. The paired analysis of HCT serum concentrations among women from the Costa Rican study with repeated samples found that HCT concentrations decreased significantly from the first to the second sample. The prenatal exposure via the placenta could explain the decrease in HCT serum concentrations in the Costa Rica mothers at their 2^nd^ visit. In contrast, our multivariable regression models found no clear effect of gestational age. This seeming difference makes sense in the context of the two different analyses. The multivariable regression models were stratified by sample number and had limited variability in gestational age. Furthermore, the paired analysis allows to identify smaller within-person changes than the cross-sectional multivariate analysis of the regression models [39].

Serum from pregnant women in Costa Rica showed about four times higher HCT concentrations compared to Swedish women. This difference may be explained by the fact that most of the pregnant women from the Costa Rican study lived near banana plantations where aerial spraying of chlorothalonil was common. The Swedish women may be exposed to lower levels of chlorothalonil from other sources, for example, diet. Among women from the Costa Rican study, residential distance to banana plantations was inversely associated with HCT serum concentrations. Similar findings have been found for urinary metabolite concentrations of the fungicides mancozeb and pyrimethanil, which are also aerially sprayed on bananas [32, 36, 40]. Chlorothalonil has been highly used on bananas in Costa Rica [41], and aerial spraying of pesticides is common [36]. Indeed, all women in the study lived in the banana plantation region (Fig. S4) [35, 36]. In addition, women who worked in agriculture tended to have higher HCT serum concentrations than women who did not. Similar results were found for mancozeb and chlorpyrifos exposure in the same population [32, 36, 40]. Only a small percentage of the women worked in agriculture during pregnancy (8%), yet of those who did, the vast majority worked on banana plantations [35]. In contrast, having a partner working in agriculture was associated with lower serum HCT – a finding we cannot explain.

In addition to environmental and occupational exposure, Costa Rican women may be exposed to higher residuals in food as compared to Swedish women. A cross-sectional survey from 2016 among smallholder farmers from Costa Rica (*n* = 300) found that chlorothalonil was the most applied pesticide on potatoes, carrots, and coriander, among other crops [42]. Additionally, chlorothalonil has been found in high levels in the environment in Costa Rica. A screening of superficial surface waters between 2009 and 2019 found chlorothalonil in concentrations that exceeded international regulations [43]. Chlorothalonil was also the most prevalent pesticide detected in air and soil in samples from remote montane forest in 2009, indicating the occurrence of atmospheric transport [44, 45].

The fact that we found HCT in the general population in Sweden was unexpected, as the use of chlorothalonil has been discontinued for agricultural use in Sweden since 1991 [9]. But chlorothalonil is still allowed, both for agricultural and other applications, in many countries outside the EU, such as those of Southeast Asia [46] as well as the United States [4, 47] and Costa Rica [27], and was only recently banned EU-wide in 2019 regarding agricultural purposes [8]. The general Swedish population might be exposed to chlorothalonil and/or the transformation product HCT via different exposure pathways, e.g. from paints, contaminated food or drinking water, or long-range transport through the global atmosphere. The seasonal trend of HCT serum concentrations may indicate consumption of chlorothalonil-contaminated batches of fruits and vegetables imported mainly in spring and summer. Chlorothalonil is indeed used in production of various fruit and vegetables, and both chlorothalonil and HCT have been identified as dominant compounds in the total pesticide residue on cereals, peanuts, tomatoes, and potatoes [6, 48]. The European Food Safety Authority (EFSA) recently derived maximum residue levels of chlorothalonil in several foods, with 0.02 mg/kg for chlorothalonil and 0.01 mg/kg for HCT (SDS-3701) in bagged bananas from Latin America [48]. Scandinavia is heavily dependent on imports for both fruit and vegetables, with the largest tonnages for bananas, oranges, and apples, followed by tomatoes, lettuce, and cucumbers [49]. The biggest world importer of bananas is the EU, with most banana imports during spring and summer from Latin America [50]. According to the EU monitoring report from 2021, chlorothalonil was repeatedly found in randomly sampled food grown in the EU at levels exceeding the MRLs, e.g. in spinach and lettuce [51]. The Swedish Food Agency published during the relevant years matching our samples (1997–2015) findings of chlorothalonil in cucumbers [52].

Drinking water might also be a possible exposure source. A recent screening of Swiss groundwater detected eight different chlorothalonil transformation products [53]. While HCT (R182281) was not measured, its transformation product 2,4,5-Trichloro-3-cyano-6-hydroxybenzamide (R611968) was detected [53]. In Sweden, chlorothalonil has not been found within the Swedish regional aquatic monitoring of pesticides yet, again, chlorothalonil transformation products have not been measured [9]. Other exposure scenarios may include the use of chlorothalonil e.g. in paints, possibly contaminating the groundwater. However, chlorothalonil was not detected in a large monitoring study of environmental samples in surface water in 2008 [9].

Chlorothalonil belongs to the family of organochlorine pesticides, which usually have a long biological and environmental half-life, as they are highly soluble in the lipid compartments of living organisms and do not degrade in the environment [54]. This might lead to continuous chlorothalonil (or HCT) exposure of the general population, even after chlorothalonil has been banned, as is known for other organochlorine pesticides [54]. International trade could lead to the “off-loading” of chlorothalonil emissions and associated exposures, as has been demonstrated for PBDE [55]. This might be the case when Sweden imports chlorothalonil-treated crops; associated chlorothalonil exposure is off-loaded to communities in Costa Rica. These are challenges for the achievement of more sustainable consumption and production patterns, two goals that are included in the 17 *Sustainable Development Goals* established by the United Nations in 2015 [56].

Our data suggest that exposure to HCT may be more widespread than previously known and that the women living in agricultural areas in low- and middle-income countries are more highly exposed than women in high-income countries. Further studies are needed to investigate possible sources and health effects associated with exposure, as well as elimination studies calculating the half-life of chlorothalonil and HCT in humans. In addition, early-life exposure needs to be explored, as both the Swedish and Costa Rican women had decreased HCT concentrations with increased parity. Further studies should investigate both transplacental and lactational transfer to clarify the mechanisms of early-life exposure.

### Supplementary Information

Supplementary Information

Reporting Checklist

## Data Availability

Raw data are available from the corresponding author on reasonable request.
