# Correction to: Detection of the fungicide transformation product 4-hydroxychlorothalonil in serum of pregnant women from Sweden and Costa Rica

**DOI:** 10.1038/s41370-023-00604-3

**Published:** 2023-09-23

**Authors:** Annette M. Krais, Berna van Wendel de Joode, Emelie Rietz Liljedahl, Annelise J. Blomberg, Anna Rönnholm, Marie Bengtsson, Juan Camilo Cano, Jane A. Hoppin, Margareta Littorin, Christel Nielsen, Christian H. Lindh

**Affiliations:** 1 Division of Occupational and Environmental Medicine, Department of Laboratory Medicine, Lund University, Lund, Sweden; 2 Infants’ Environmental Health Study (ISA), Central American Institute for Studies on Toxic Substances (IRET), Universidad Nacional de Costa Rica, Heredia, Costa Rica; 3 Center for Human Health and the Environment, North Carolina State University, Raleigh, NC USA; 4 Department of Biological Sciences, North Carolina State University, Raleigh, NC USA; 5 Division of Clinical Pharmacology, Pharmacy and Environmental Medicine, Department of Public Health, University of Southern Denmark, Odense, Denmark

Correction to: *Journal of Exposure Science & Environmental Epidemiology* 10.1038/s41370-023-00580-8, published online 20 July 2023

The original version of this article unfortunately contained a small error in the chemical structure of Fig. 1.

The original article has been corrected.

The corrected Fig. 1 is presented below:
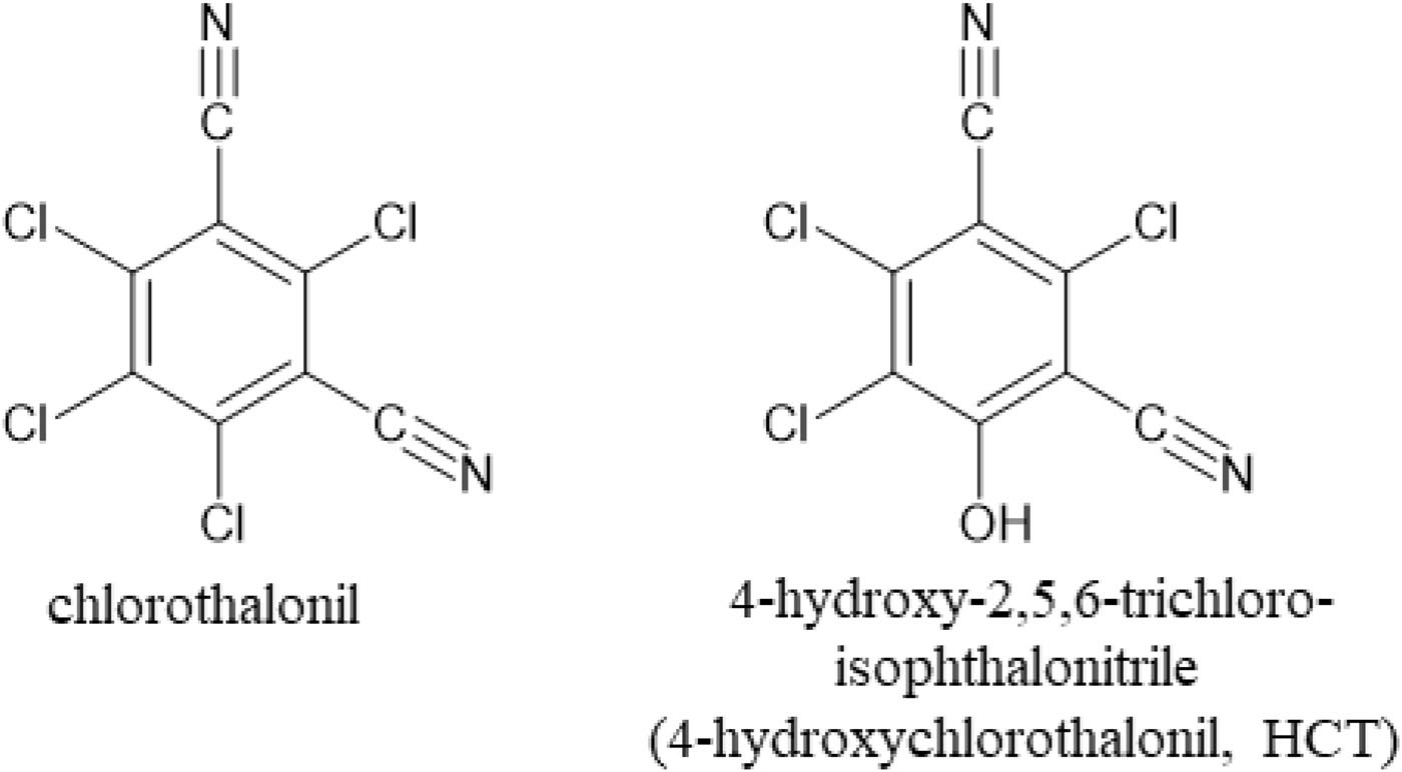


Fig. 1 Chemical structures of chlorothalonil and 4-hydroxychlorothalonil (HCT).

