# Supplementary material for: Detection of the fungicide transformation product 4-hydroxychlorothalonil in serum of pregnant women from Sweden and Costa Rica
Source: J Expo Sci Environ Epidemiol. 2023 Jul 20;34(2):270–7. doi: 10.1038/s41370-023-00580-8 (PMC11142910; doi:10.1038/s41370-023-00580-8)
Supplement: Supplementary file 1 — Supplementary Information [file 41370_2023_580_MOESM1_ESM.docx]

**Supplementary Information**

# **Material and methods**

## Quantitative analysis of HCT in serum

## *Chemicals, reagents, and materials*

The analytical standard solution 4-hydroxychlorothalonil (HCT) was purchased from Dr. Ehrenstorfer (Augsburg, Germany). Acetonitrile, methanol (Supelco®, LiChrosolv®, hypergrade for LC-MS) and ammonium acetate (EMSURE® ACS Reag. PhEur, for analysis) were from Merck (Darmstadt, Germany). β-Glucoronidase from *E. coli* K12 was obtained from Roche Diagnostics (Mannheim, Germany). Water from a Milli-Q® Integral 5 system (Millipore, Billerica, MA, USA) was used. The 96-well carrier plates with 1.5 ml flat bottom glass inserts, and 96-plug solid sealing mats (Molded Flat Mat PTFE/Silicone White Liner) were all from the Multi-Tier™ MTP System Topas (J.G. Finneran Associates, Vineland, NJ, USA). The 96-well carrier plates (Deep, PP, 1.3 ml) with 0.5 ml conical glass inserts, and 96-plug injectable sealing mats (Blue CapMat with pre-cut PTFE/Silicone Septa) were all from WHEATON® MicroLiter Plate Sampling System™ (DWK Life Sciences, Millville, NJ, USA).

## *Instrumentation*

Quantitative analysis was conducted using a triple quadrupole linear ion trap mass spectrometer equipped with TurboIonSpray sources (QTRAP® 5500+, AB Sciex, Framingham, MA, USA) coupled to a liquid chromatography system (UFLCXR, Shimadzu Corporation, Kyoto, Japan; LC-MS/MS). Nitrogen was used as nebulizer, auxiliary, curtain, and collision gas. The MS analyses were carried out using selected reaction monitoring (SRM) in negative ion mode. The SRM conditions are shown in Table S1. All data acquisition was performed using Analyst 1.7.2 software and data processing was performed using Multiquant 3.0.1 (AB Sciex, Framingham, MA, USA).

**Table S1.** Analytical details for the quantification of 4-hydroxychlorothalonil (HCT). The table includes mass transitions (Q1 and Q3 masses in Dalton (Da)), dwell time (msec), collision energy (CE) in electron volt (eV), and declustering potential (DP) in volt (V).

| **ID** | **Q1 (Da)** | **Q3 (Da)** | **Dwell time (msec)** | **CE (eV)** | **DP (V)** |
| --- | --- | --- | --- | --- | --- |
| HCT (1) | 245.0 | 175.0 | 8.0 | -38 | -80 |
| HCT (2) | 245.0 | 35.0 | 8.0 | -68 | -80 |
| HCT (3) | 245.0 | 210.0 | 8.0 | -34 | -80 |
| HCT (4) | 245.0 | 182.0 | 8.0 | -40 | -80 |

## *Calibration standards, chemical blanks, and quality control samples*

Stock solutions were prepared by dissolving accurately weighed amounts of HCT in acetonitrile. Standard solutions were prepared by further dilution of the stock solutions in acetonitrile at concentrations of 400, 200, 100, 20, 10, 2, and 0 µg/L. For the calibration standards, serum matrix (human serum from healthy volunteers) was used and prepared in the same way as the samples, except for the addition of 25 µL of diluted standard solutions (i.e., after the digestion step described in the sample preparation procedure). Two in-house prepared reference samples were used for quality control (QC1 and QC2) by pooling samples from individuals from our laboratory, with low known levels of HCT. The two quality control samples, four chemical blanks (water), and calibration standards were included in each sample batch (96-well plate). No internal standard was available for HCT at the time of method development. Recently, a ^13^C_2_^15^N_2_-labelled HCT standard has become available (ASCA, GmbH Angewandte Synthesechemie, Berlin, Germany).

## *Sample preparation of serum samples*

Samples were stored at -20°C until analysis. The thawed and homogenized samples were prepared in 96-well carrier plates with 1.5 mL flat bottom glass vials. 10 µl of β-glucuronidase and 10 µL of a 1 M ammonium acetate buffer (pH 6.5) were added to 100 µL of serum sample. The 96-well plate was covered with a sealing mat, and samples were digested and mixed at 37°C for 90 min. Thereafter, 25 µL of acetonitrile were added to each sample, except for calibration curve matrix samples, where 25 µL of the diluted standard solution were added instead. To precipitate the proteins, 200 µl of acetonitrile were added to all samples, the 96-well plate sealed again, followed by vigorous shaking for 30 min. The samples were thereafter centrifuged at 2600 × g for 10 min. The supernatant (200 µL) was transferred to a new 96-well plate carrier with 0.5 ml conical glass vials for analysis, covered with an injectable sealing mat, and again centrifuged at 3000 × g for 10 min before analysis.

## *Analysis of HCT*

An aliquot of 5 µL of the supernatant was analyzed with LC-MS/MS. A Genesis Lightning C18 column, 4 µm, 50 × 2.1 mm (Avantor, VWR International, Lutterworth, UK) was used for the analysis before the injector to filter the mobile phases from contaminating substances. The analytical column was a Gemini NX-C18, 110Å, 3 µm, 100 × 2.0 mm (Phenomenex, Torrance, CA, USA). The mobile phases were 5 mM ammonium acetate in water (A) and methanol (B). The mobile phase was kept at 5% B for 1 min after injection. A gradient was then applied up to 95% B for 4 min, where it was kept for 1 min. The column was then conditioned at 5% B for 2.1 min. The columns and LC pumps were connected through a diverter valve, and the column effluent was diverted to the MS between 3.9 and 4.8 min. The flow rate was 0.6 mL/min, the column was maintained at 55°C and the total analytical run-time was 8.2 min. The analysis was performed in negative ionization mode, with the declustering potential (DP) of -80V, ion source temperature at 600°C and ion spray voltage at -4500V.

***Quantification of serum samples***

The final concentrations of the calibration standards after sample preparation were 100, 50, 25, 5, 2.5, 0.5, and 0 µg/L. Serum concentrations were determined using the peak area of the analyte compared to those of the standard curve. The quantifier and qualifier transitions are shown in Table S1. The quantitative analysis of HCT in this study was performed using the transitions m/z 245/175 and 245/35. The final concentrations were only reported if the ratio of the transitions were consistent with the standards (± 20% and levels >0.1 µg/L).

## *Quality control, limit of detection, and precision of serum samples*

The limit of detection (LOD) was determined to 0.1 µg/L and was defined as three times the standard deviation of the concentration corresponding to the peak at the same retention time as HCT in the chemical blanks (n = 128). Equally, the limit of quantification (LOQ) was determined as 0.4 µg/L and defined as ten times the standard deviation. The results of the quality control samples were used to calculate the between-run precision of the method (Table S2), determined as the coefficient of variation (CV) of the quality control samples. The quality control samples were analyzed with every analytical batch in duplicates. The between-batch precision was determined by comparing the duplicate analyses of samples from the exposure studies. The samples were prepared and analyzed in separate analytical batches. After analysis, the concentrations were grouped into three different ranges, and the averages were used to determine the CV (Table S2). The formula below was used to calculate the standard deviation *s*, where *x_1_* and *x_2_* are the results of the separate aliquots analyzed, and *n* is the number of samples.

$$s=\sqrt{\frac{1}{2n}\cdot\sum_{i=1}^{i=n} (x_{1}-x_{2})_{n}^{2}}$$

A sub-set of 70 serum samples have been re-analyzed with internal standard. The concentration of HCT in the samples shows an excellent correlation (Figure S1 A). To assess the inter-individual matrix effect, we have plotted the response for the internal standard at the transition m/z 251/188 in 200 real serum samples from another cohort. The response is shown in Figure S1 B.

**Figure S1:** A. Re-analysis of 70 serum samples quantified with and without internal standard. B. Response for the internal standard in 200 real serum samples evaluated for the transition m/z 251/188.


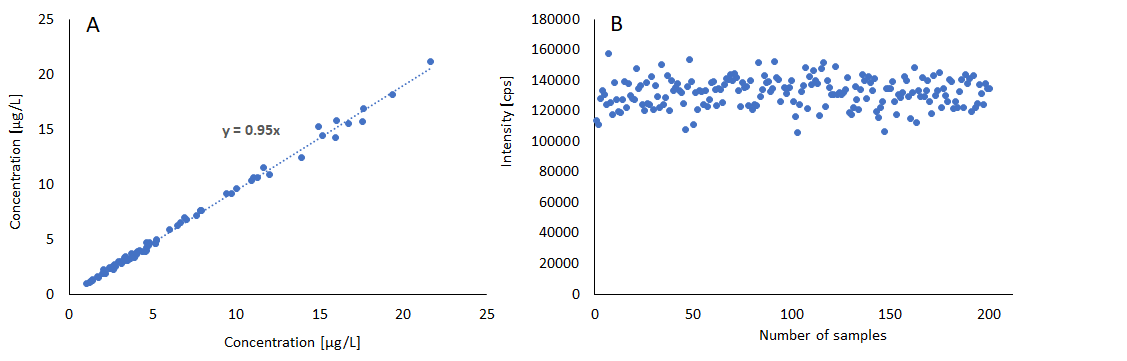


**Table S2.** Between-batch precision and between-run precision. Between-batch precision was calculated with mean values (MV) and coefficient of variation (CV) in three different concentration ranges, after several analytical runs (*N*). Between-run precision was calculated with the CV of the QC1 and QC2 samples.

|  | **Samples** | **mean (µg/L)** | **CV (%)** | ***N*** |
| --- | --- | --- | --- | --- |
| Between-batch precision | MV 1 | 6.8 | 7.3 | 237 |
|  | MV 2 | 15 | 7.4 | 238 |
|  | MV 3 | 32 | 11 | 238 |
| Between-run  precision | QC 1 | 5.2 | 13 | 39 |
|  | QC 2 | 3.4 | 14 | 39 |

**Results**

**Method validation of HCT**

**Figure S2:** A real serum sample with a quantitated level of 1 µg/L with the transitions m/z 245/35 (A) and m/z 245/175 (B). LOQ of the method was 0.4 µg/L serum.

**
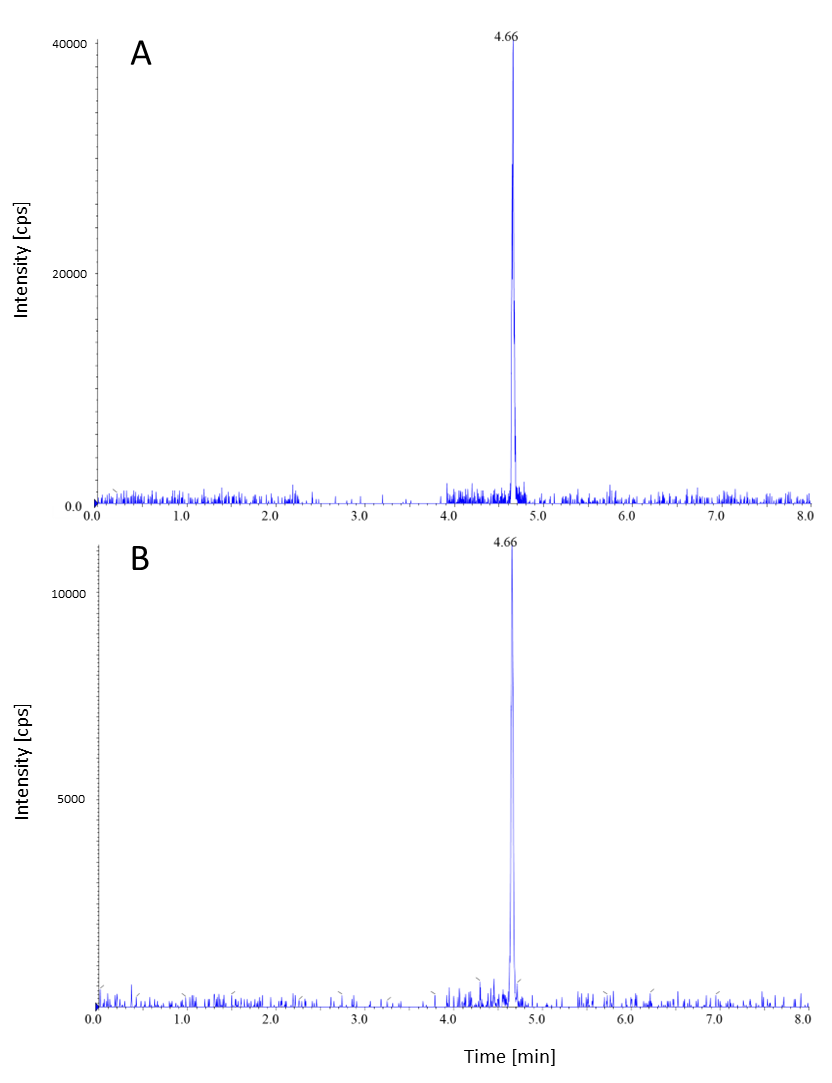
**

**HCT serum concentrations in Sweden**


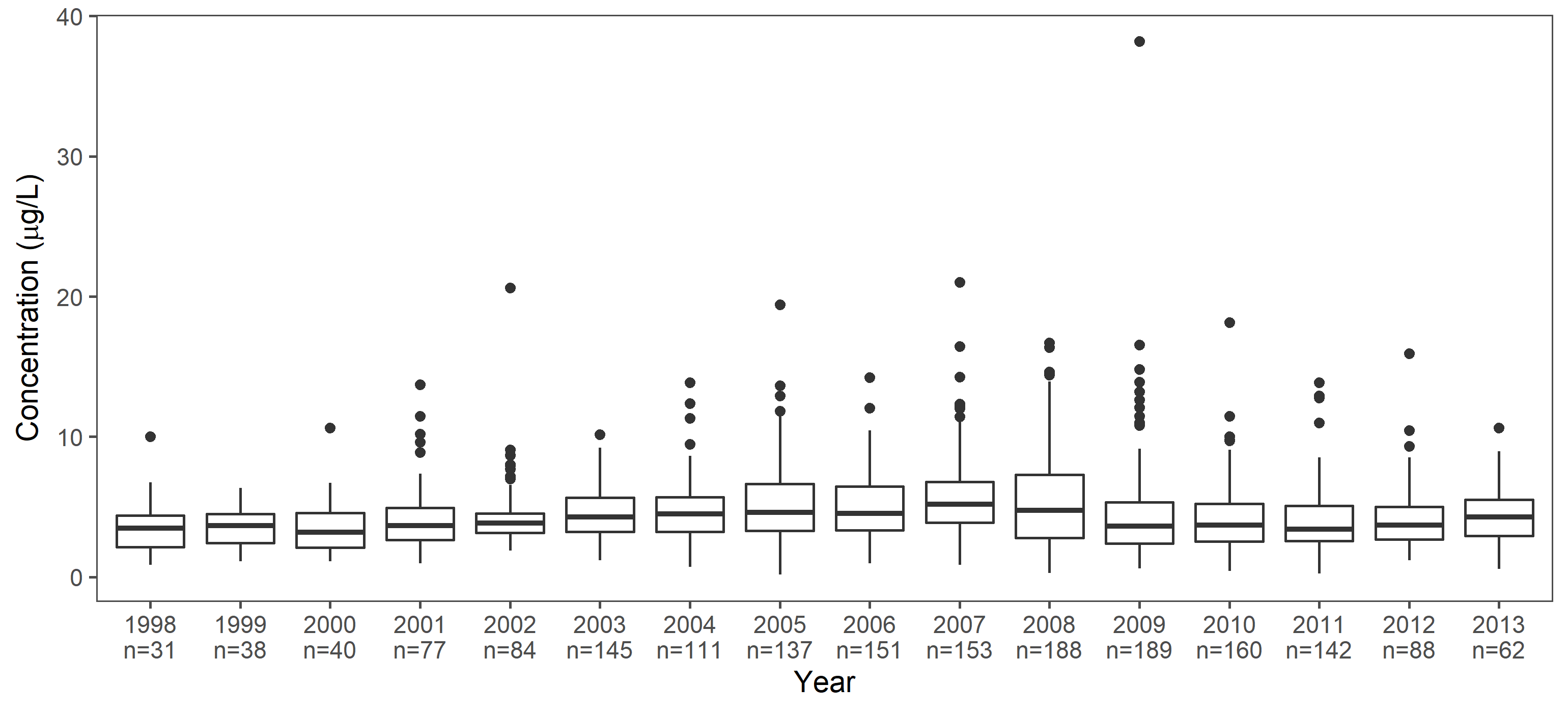


**Figure S3:** Boxplot of 4-hydroxychlorothalonil (HCT) serum concentrations (µg/L) in 1796 pregnant Swedish women by sampling year. Samples (n = 12) from years 1997, 2014, and 2015 are not included in the boxplot, due to small sample numbers (see Table S3).

**Table S3:** Summary of 4-hydroxychlorothalonil (HCT) serum concentrations (µg/L) in 1808 pregnant Swedish women by season, with number of recruited women (*N*), median values with IQR, and range.

| Season | *N* | Median (IQR) | Range | *P* (Kruskal Wallis)^a^ | |
| --- | --- | --- | --- | --- | --- |
| Winter | 490 | 4.1 (3.0−5.8) | 0.7−16 | <0.001 |  |
| Spring | 396 | 4.8 (3.5−6.9) | 0.8−38 |  |  |
| Summer | 425 | 4.2 (2.8−5.9) | 0.3−19 |  |  |
| Autumn | 497 | 3.6 (2.5−4.9) | 0.2−17 |  |  |

^a^Pairwise comparison between seasons using Mann-Whitney with Bonferroni correction were all *P* < 0.001 except for winter *vs* summer (*P* = 0)

**Table S4.** Results from simple regression models presenting percentage change in 4-hydroxychlorothalonil (HCT) serum concentrations associated with sociodemographic, pregnancy, environmental and occupational variables, among pregnant women from the Costa Rican cohort (2010−2011).

| **Variables** | **First samples, n = 393** | **Second samples, n = 239** |
| --- | --- | --- |
|  | ***% Change HCT (95% CI)*** | ***% Change HCT (95% CI)*** |
| **Maternal age (years) (tertiles)**  ≥19 – 21 vs. <19  ≥22 – 27 vs. <19  ≥28 vs <19 | 14 (-6, 38)  32 (10, 59)  31 (8, 58) | 25 (0, 55)  24 (0, 53)  13 (-9, 40) |
| **Parity**  1 vs. 0  2 vs. 0 | 3 (-12, 22)  0 (-15, 17) | 4 (-14, 25)  -8 (-24, 11) |
| **1 week increase in gestational age** | -0.54 (-1.39, 0.31) | -0.56 (-1.85, 0.74) |
| **Residential distance to banana plantations (m) (tertiles)**  ≥90 – 372 vs. <90  ≥373 vs. <90 | 3 (-12, 22)  -18 (-31, -4) | -2 (-18, 18)  -26 (-38, -10) |
| **Woman works in agriculture** | 35 (6, 73) | 25 (-5, 64) |
| **Partner works in agriculture** | -14 (-25, -2) | -10 (-23, 5) |
| **Relatively dry season (Feb-Apr or Oct-Nov)** | 9 (-5, 25) | -3 (-17, 14) |

**4-hydroxychlorothalonil (HCT) serum concentrations and distance to banana plantations in the Costa Rican cohort**


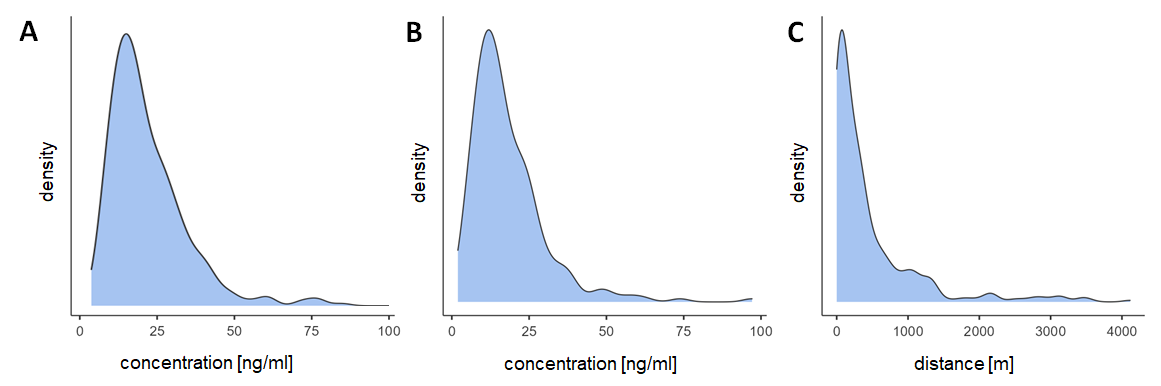


**Figure S4.** Distribution of 4-hydroxychlorothalonil (HCT) concentrations in serum from pregnant women in Costa Rica from the first sampling (A, n = 393) and the second sampling (B, n = 239), as well as their residential distance to banana plantations (C, n = 393).
